# Supplementary material for: Synthesis of Novel N-Heterocyclic Carbene-Ruthenium (II) Complexes, “Precious” Tools with Antibacterial, Anticancer and Antioxidant Properties
Source: Antibiotics (Basel). 2023 Apr 1;12(4):693. doi: 10.3390/antibiotics12040693 (PMC10135378; doi:10.3390/antibiotics12040693)
Supplement: Supplementary file 1 [file antibiotics-12-00693-s001.zip › antibiotics-2300846-supplementary.pdf]

Supplementary Materials

Synthesis of Novel *N*-Heterocyclic Carbene-Ruthenium (II) Complexes, "Precious" Tools with Antibacterial, Anticancer and Antioxidant Properties

Jessica Ceramella [1,†], Rubina Troiano [2,†], Domenico Iacopetta [1], Annaluisa Mariconda [3,*], Michele Pellegrino [1], Alessia Catalano [4], Carmela Saturnino [3], Stefano Aquaro [1], Maria Stefania Sinicropi [1,*] and Pasquale Longo [2]

[1] Department of Pharmacy, Health and Nutritional Sciences, University of Calabria, Via P. Bucci, 87036 Arcavacata di Rende, Italy; jessica.ceramella@unical.it (J.C.); domenico.iacopetta@unical.it (D.I.); michele.pellegrino@unical.it (M.P.); stefano.aquaro@unical.it (S.A.)

[2] Department of Chemistry and Biology, University of Salerno, Via Giovanni Paolo II, 132, 84084 Fisciano, Italy; rutroiano@unisa.it (R.T.); plongo@unisa.it (P.L.)

[3] Department of Science, University of Basilicata, Viale dell'Ateneo Lucano 10, 85100 Potenza, Italy; carmela.saturnino@unibas.it

[4] Department of Pharmacy-Drug Sciences, University of Bari "Aldo Moro", 70126 Bari, Italy; alessia.catalano@uniba.it

* Correspondence: annaluisa.mariconda@unibas.it (A.M.); s.sinicropi@unical.it (M.S.S.)

† These authors contributed equally to this work.

List of Contents

Characterization of **L1**……………………………………………………S1

Characterization of **L2**……………………………………………………S4

Characterization of **L3**……………………………………………………S7

Characterization of **RANHC-I**………………………………………………S10

Characterization of **RANHC-V**………………………………………………S13

Characterization of **RANHC-VI**………………………………………………S16

Characterization of **RANHC-II**………………………………………………S19

Characterization of **RANHC-III**………………………………………………S22

Characterization of **RANHC-IV**………………………………………………S27

Anticancer activity of the studied Ru-NHC complexes (**RANHC-I-VI**)……………S30

MIC results of the Ru-NHC complexes (**RANHC-I-VI**)……………………………S30

Radical scavenging ability against DPPH and ABTS radicals…………………………S31

^{1}H-NMR N-Methyl, N′-(2-methoxy-2-phenyl)ethyl imidazolium iodide (L1)

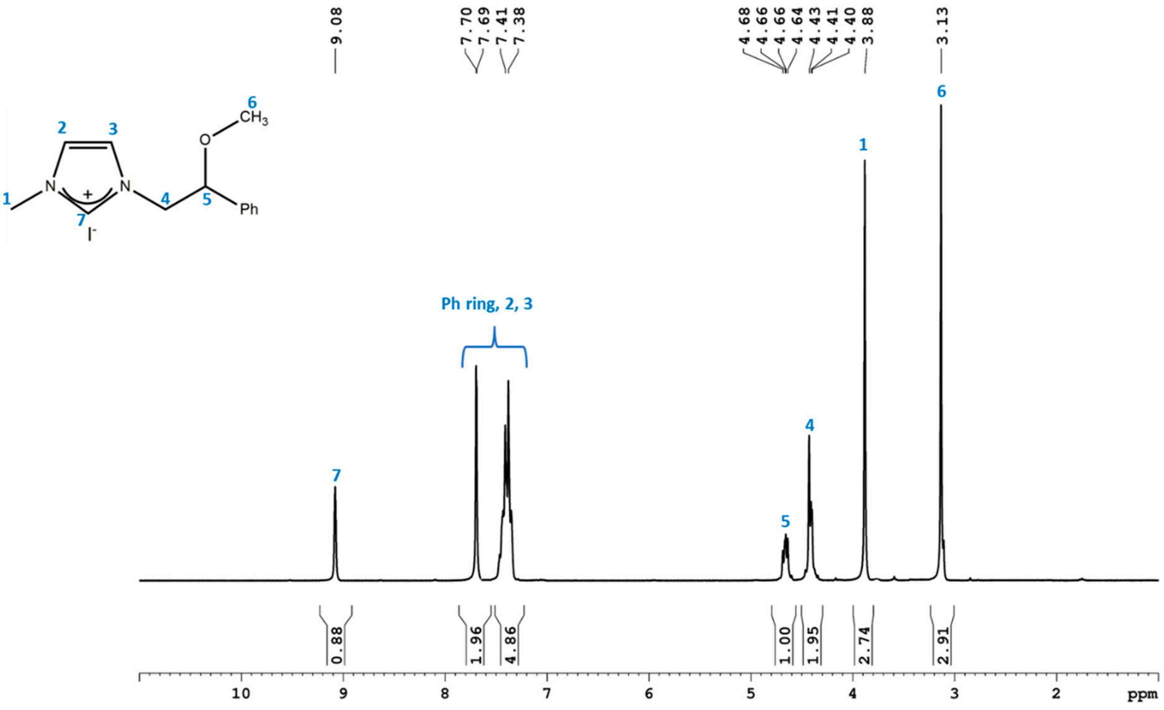

Figure S1: *^{1}H NMR spectrum of N-Methyl, N′-(2-methoxy-2-phenyl)ethyl imidazolium iodide (**L1**).*

^{1}H-NMR (ppm, DMSO-d_6, 250 MHz): δ 9.08 (s, NC***H***N, 1H), 7.70–7.38 (m, aromatic hydrogens and NC***H***C***H***N, 7H), 4.66 (m, C***H***OCH_3, 1H), 4.41 (m, NC***H***$_2$CHOCH_3, 2H), 3.88(s, NC***H***$_3$, 3H), 3.13 (s, OC***H***$_3$, 3H).

^{13}C-NMR N-Methyl, N'-(2-methoxy-2-phenyl)ethyl imidazolium iodide (L1)

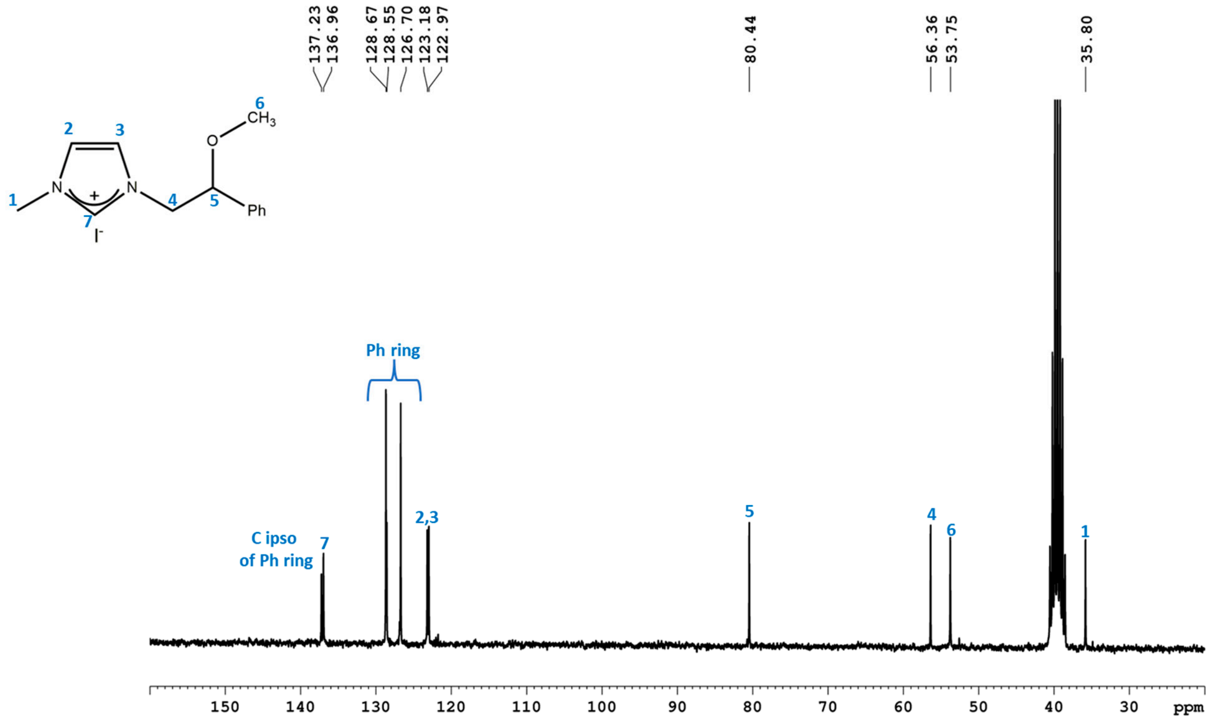

Figure S2:** ^{13}C-NMR spectrum of N-Methyl, N'-(2-methoxy-2-phenyl)ethyl imidazolium iodide **(L1).

^{13}C-NMR (ppm, DMSO-d_6, 62.5 MHz): δ 137.23 (ipso carbon of aromatic ring), 136.96 (N***C***HN), 128.67, 128.55, 126.71 (aromatic carbons), 123.18, 122.97 (NC***H***C***H***N), 80.44 (***C***HOCH$_3$), 56.36 (N***C***H$_2$CHOCH$_3$), 53.75 (O***C***H$_3$), 35.80 (N***C***H$_3$).

MALDI of N-Methyl, N′-(2-methoxy-2-phenyl)ethyl imidazolium iodide (L1)

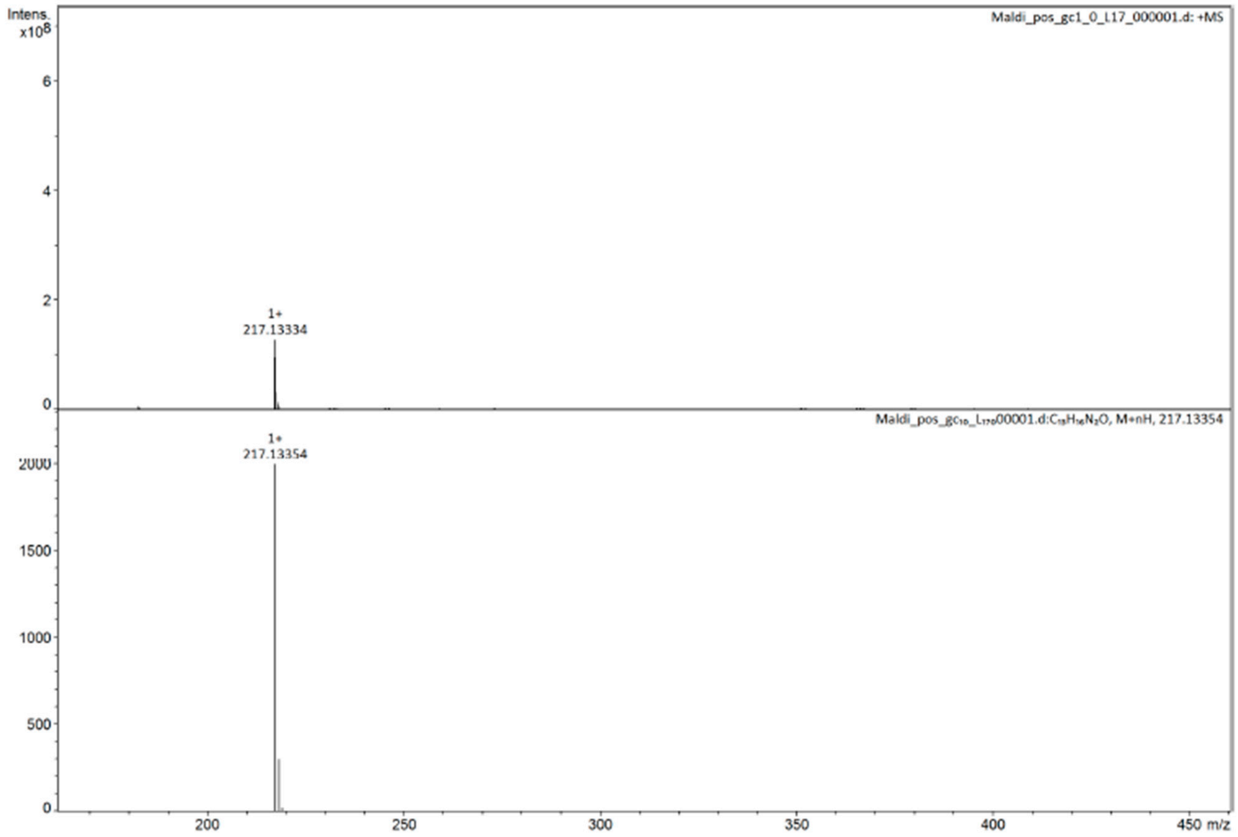

Figure S3: *MALDI of N-Methyl, N′-(2-methoxy-2-phenyl)ethyl imidazolium iodide* ***(L1)***

^{1}H-NMR N-Methyl, N′-(2-methoxy-2-phenyl)ethyl- 4,5-dichloroimidazolium iodide (L2)

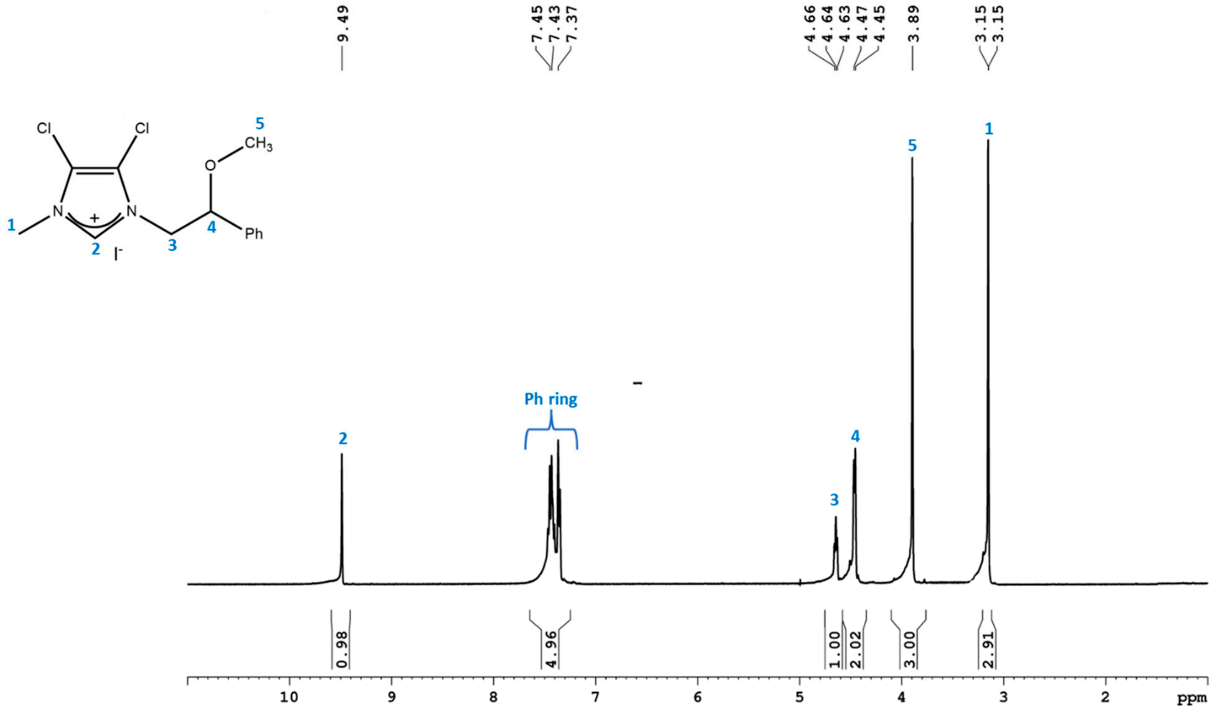

Figure S4: *^{1}H- NMR spectrum of N-Methyl, N′-(2-methoxy-2-phenyl)ethyl- 4,5-dichloroimidazolium iodide* ***(L2)***

^{1}H-NMR (ppm, DMSO-d_6, 400 MHz): δ 9.49 (s, NC***H***N, 1H), 7.45–7.37 (m, aromatic hydrogens, 5H), 4.64 (t, C***H***OCH_3, 1H), 4.46 (m, NC***H***$_2$CHOCH_3, 2H), 3.89 (s, OC***H***$_3$, 3H), 3.15 (s, NC***H***$_3$, 3H).

^{13}C-NMR N-Methyl, N′-(2-methoxy-2-phenyl)ethyl- 4,5-dichloroimidazolium iodide (L2)

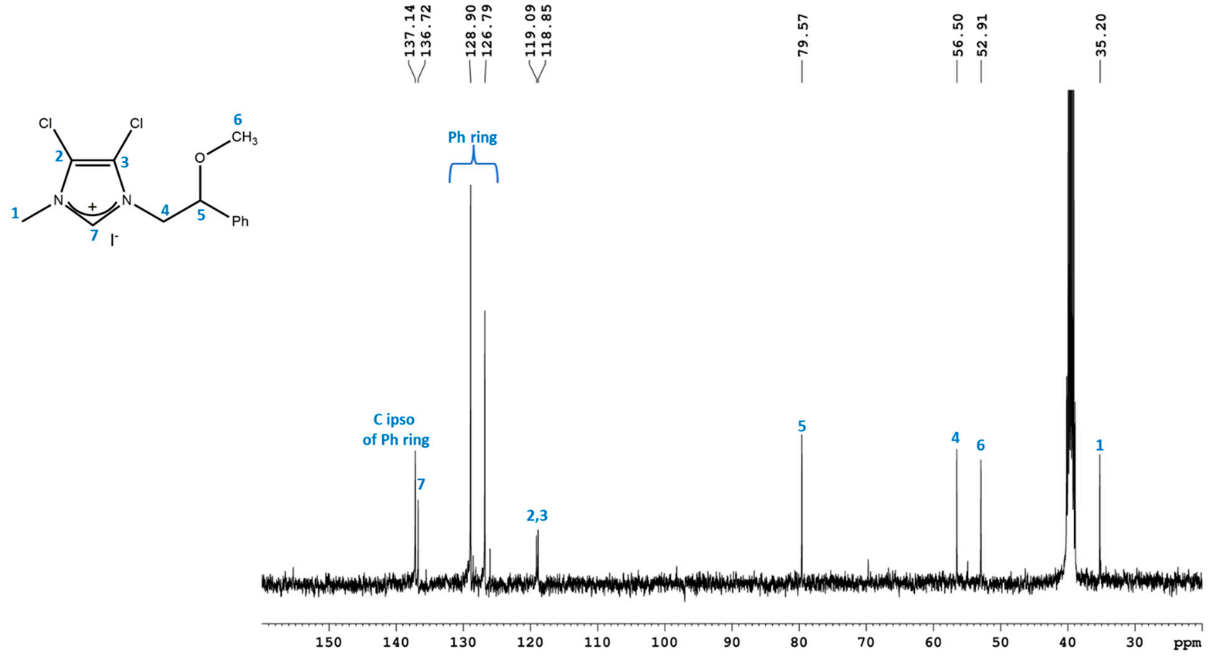

Figures S5:^{13}*C- NMR spectrum of N-Methyl, N′-(2-methoxy-2-phenyl)ethyl- 4,5-dichloro imidazolium iodide* ***(L2)***

^{13}C-NMR (ppm, DMSO-d_6, 100 MHz): δ 137.14 (ipso carbon of aromatic ring), 136.72 (N***C***HN), 128.90, 126.79, 126.00 (aromatic carbons), 119.09, 118.85 (N***C***H***C***HN), 79.57 (***C***HOCH_3), 56.50 (N***C***H_2CHOCH_3), 52.91 (O***C***H_3), 35.20 (N***C***H_3).

MALDI of N-Methyl, N′-(2-methoxy-2-phenyl)ethyl- 4,5-dichloroimidazolium iodide (L2)

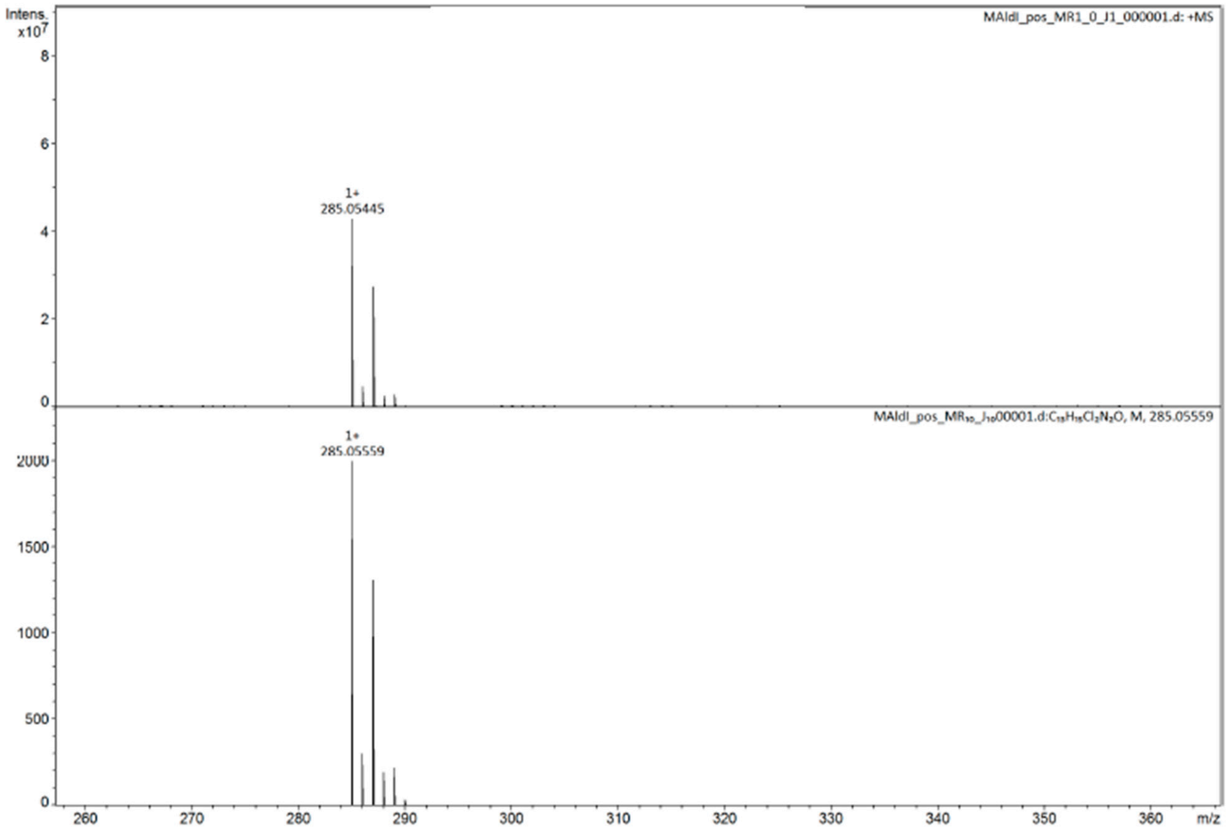

Figure S6: *MALDI of N-Methyl, N′-(2-methoxy-2-phenyl)ethyl- 4,5-dichloroimidazolium iodide* ***(L2)***

^{1}H-NMR N-Methyl, N′-(2-methoxy-2-phenyl)ethyl-benzimidazolium iodide (L3)

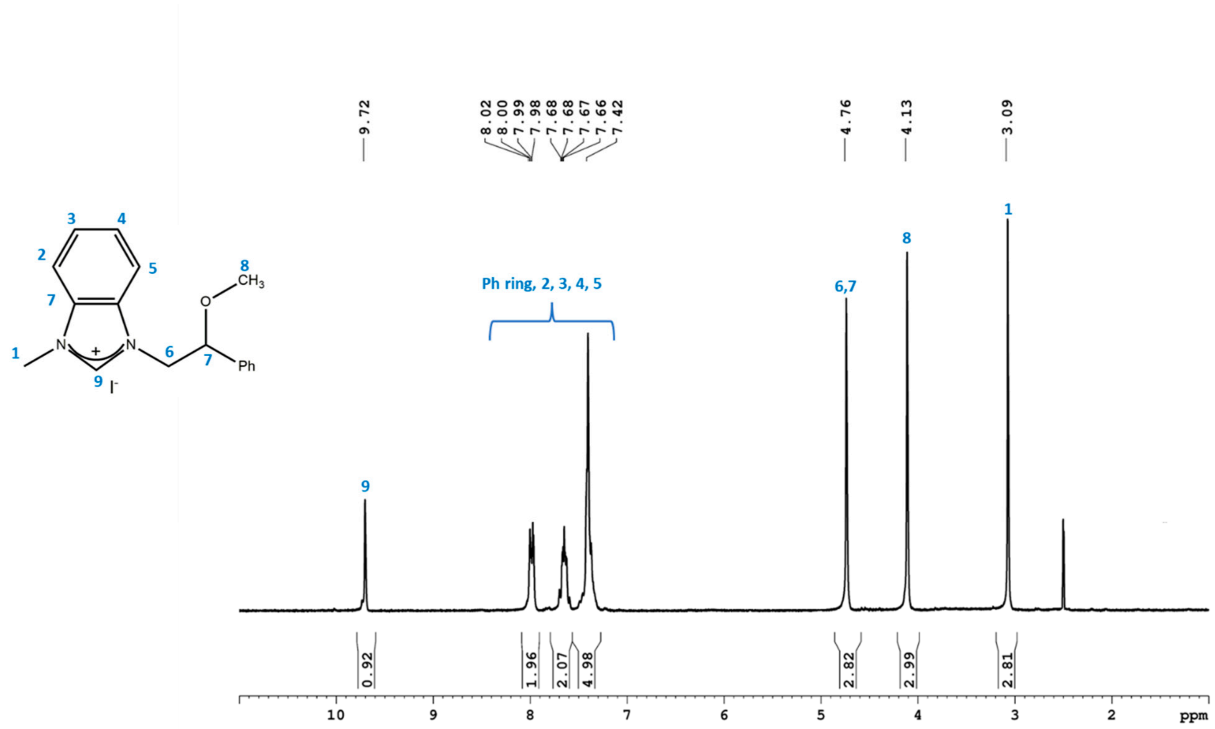

Figure S7: ^{1}H-NMR spectrum of N-Methyl, N′-(2-methoxy-2-phenyl)ethyl-benzimidazolium iodide ***(L3)***

^{1}H-NMR (ppm, DMSO-d_6, 250 MHz): δ 9.72 (s, NC*H*N, 1H), 8.02–7.42 (m, aromatic hydrogens, 9H), 4.76 (o, C*H*OCH$_3$ and NC*H*$_2$CHOCH$_3$, 3H), 4.13 (s, OC*H*$_3$, 3H), 3.09 (s, NC*H*$_3$, 3H).

^{13}C-NMR N-Methyl, N′-(2-methoxy-2-phenyl)ethyl-benzoimidazolium iodide (L3)

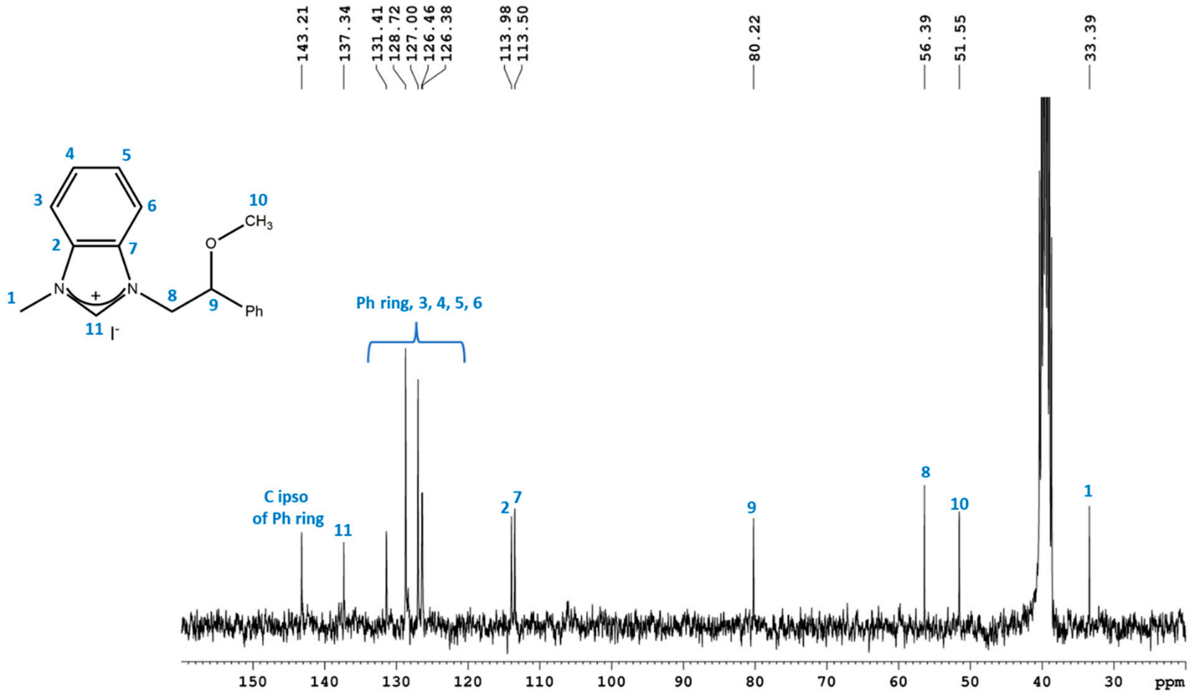

Figure S8: *^{13}C-NMR spectrum of N-Methyl, N′-(2-methoxy-2-phenyl)ethyl-benzimidazolium iodide* ***(L3)***

^{13}C-NMR (ppm, DMSO-d$_6$, 75 MHz): δ 143.21 (ipso carbon of aromatic ring), 137.34 (N***C***HN), 131.41 128.72, 127.00, 126.46, 126.38, 113.98, 113.50 (aromatic carbons), 80.22 (***C***HOCH$_3$), 56.39 (N***C***H$_2$CHOCH$_3$), 51.55 (O***C***H$_3$), 33.39 (N***C***H$_3$).

MALDI of N-Methyl, N′-(2-methoxy-2-phenyl)ethyl-benzimidazolium iodide (L3)

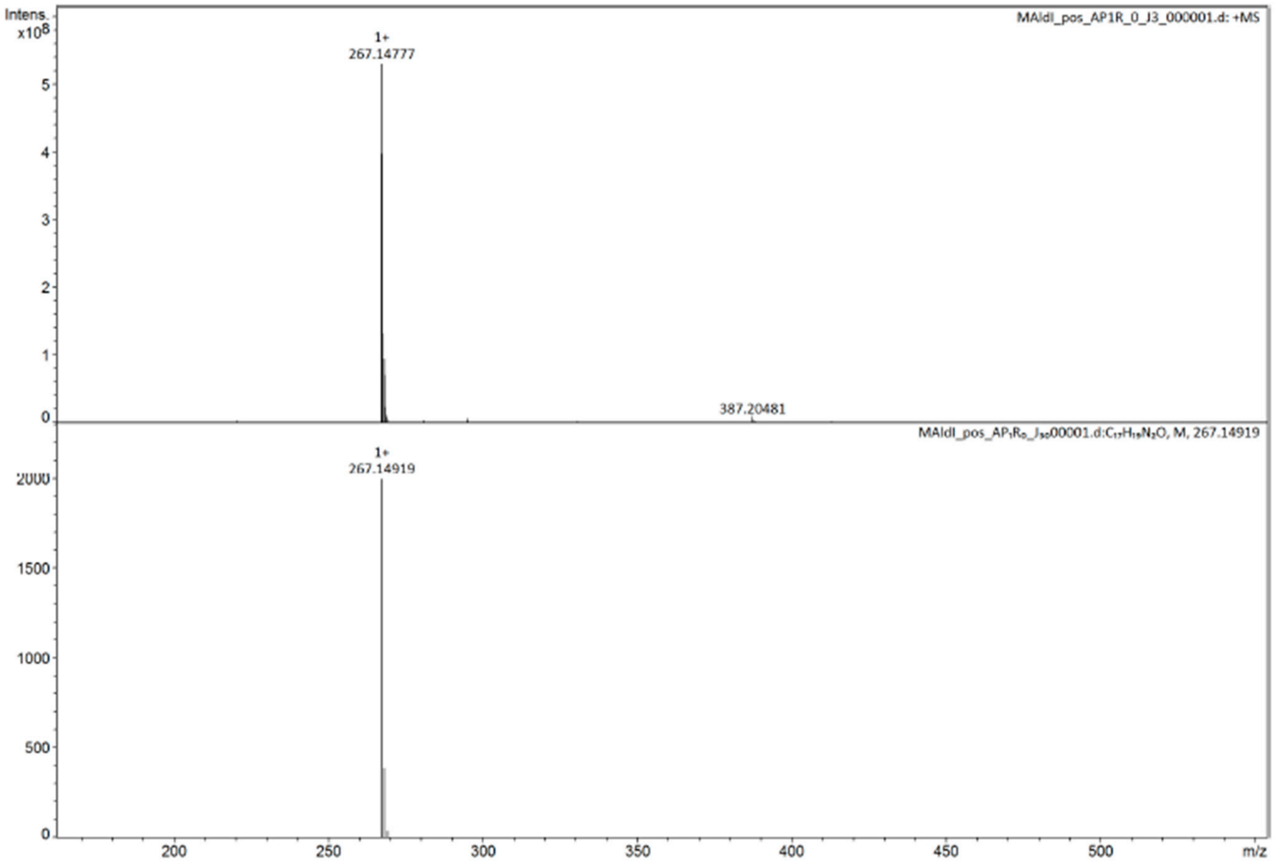

Figure S9: *MALDI of N-Methyl, N′-(2-methoxy-2-phenyl)ethyl-benzoimidazolium iodide* ***(L3)***

¹H-NMR RANHC-I

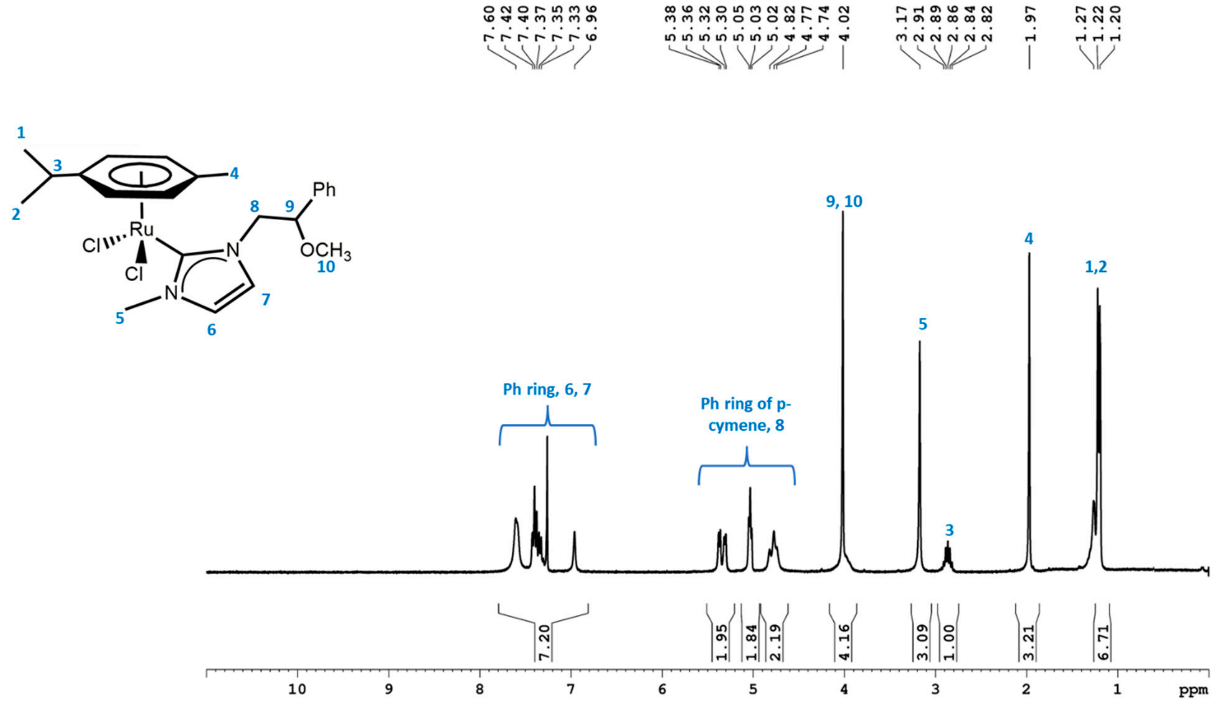

Figure S10: *¹H-NMR spectrum of* ***RANHC-I***

¹H-NMR (ppm, $CDCl_3$, 300 MHz): δ 7.60-6.96 (m, aromatic hydrogens and NC***H***C***H***N, 7H), 5.38-5.30 (dd, aromatic hydrogens of *p*-cymene, 2H), 5.03 (m, aromatic hydrogens of *p*-cymene, 2H), 4.77 (m, NC$\boldsymbol{H}_2$CHOCH_3, 2H), 4.02 (o, OC$\boldsymbol{H}_3$ and NCH_2C***H***OCH_3, 4H), 3.17 (s, NC$\boldsymbol{H}_3$, 3H), 2.86 (m, C***H***$(CH_3)_2$ *p*-cymene, 1H), 1.97 (s, C$\boldsymbol{H}_3$ *p*-cymene, 3H), 1.22 (m, CH(C$\boldsymbol{H}_3$)$_2$ *p*-cymene, 6H).

^{13}C-NMR RANHC-I

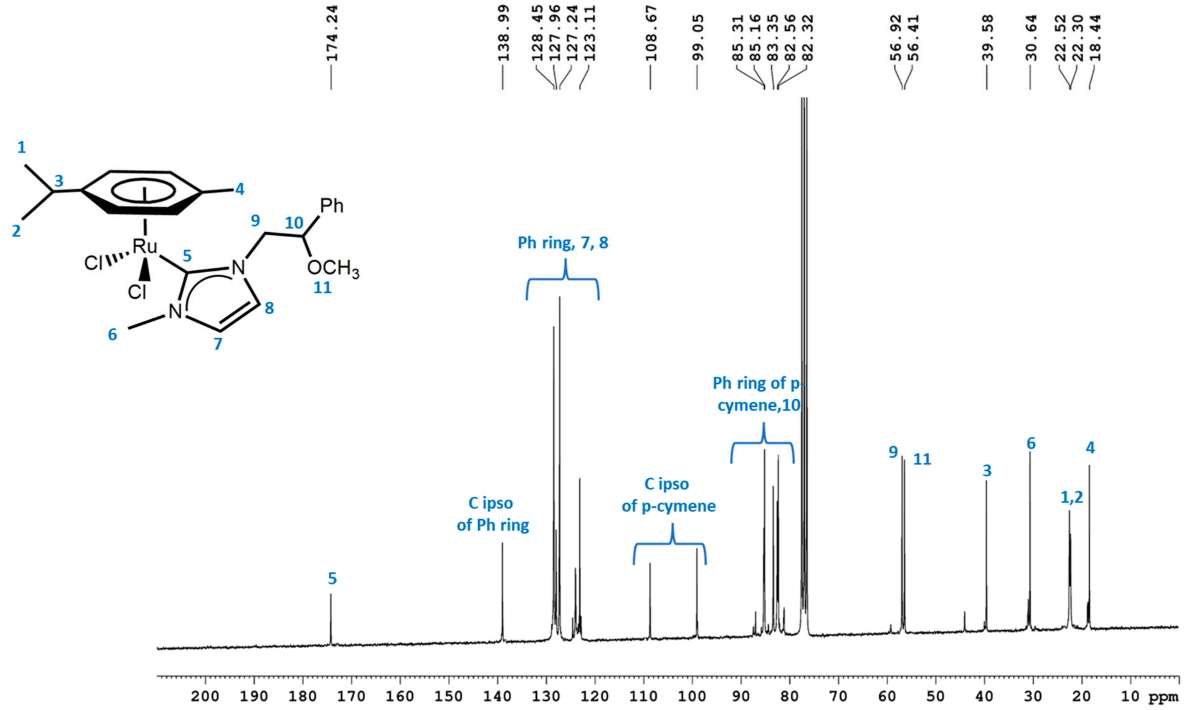

Figure S11:** ^{13}C-NMR spectrum of **RANHC-I

^{13}C-NMR (ppm, $CDCl_3$, 62.5 MHz): δ 174.24 (N***C***N), 138.99 (ipso carbon of aromatic ring), 128.45, 127.96, 127.24, 123.11 (aromatic carbons), 108.67, 99.05 (N***C***HC***H***N), 85.31, 85.16, 83.35, 82.56, 82.32 (aromatic carbon *p*-cymene, NCH_2***C***H), 56.92 (N***C***H_2CH), 56.41 (O***C***H_3), 39.58 (***C***H(CH_3)$_2$ *p*-cymene), 30.64 (N***C***H_3), 22.52, 22.30 (CH(***C***H_3)$_2$ *p*-cymene), 18.44 (***C***H_3 *p*-cymene).

ESI of RANHC-I

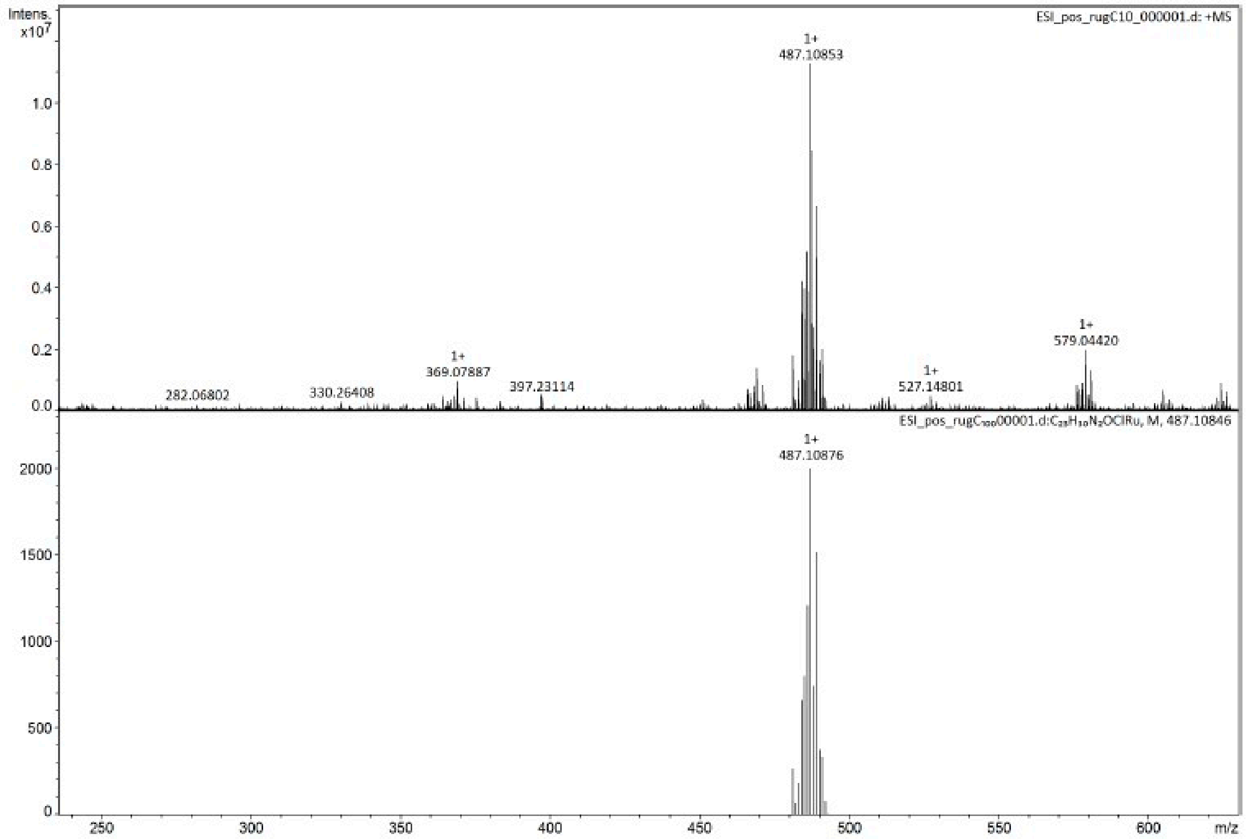

Figure S12: *ESI of* ***RANHC-I***

^{1}H-NMR RANHC-V

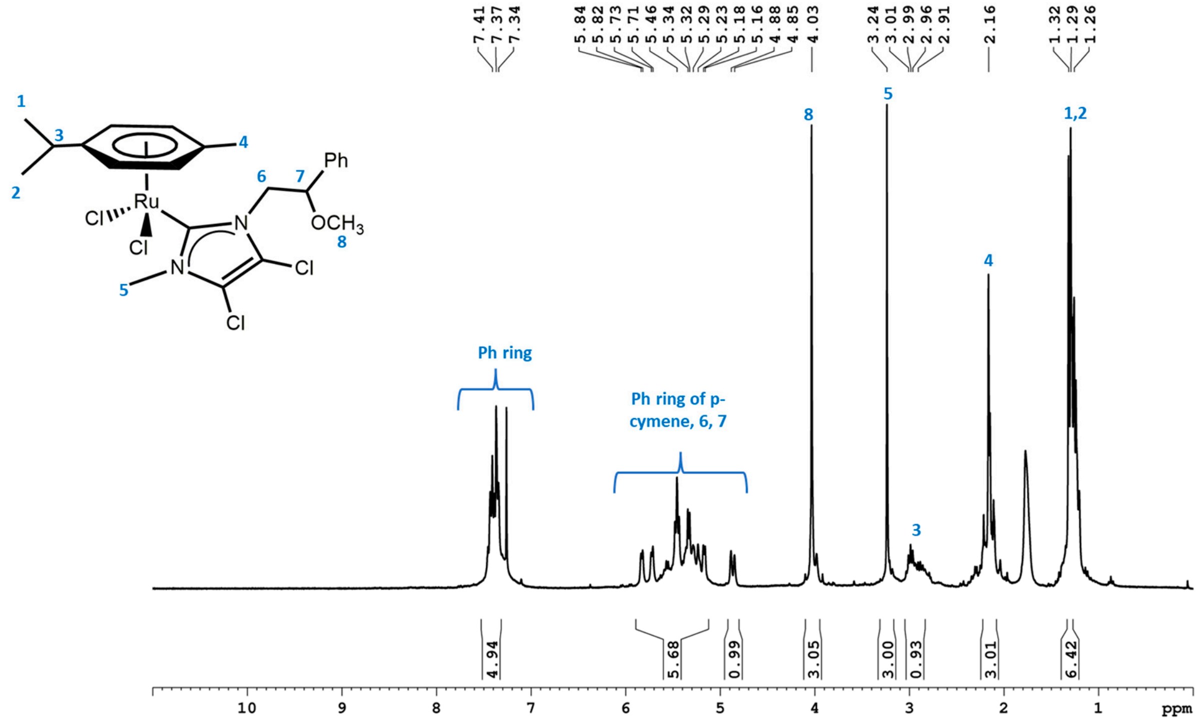

Figure S13: *^{1}H-NMR spectrum of* ***RANHC-V***

^{1}H-NMR (ppm, $CDCl_3$, 300 MHz): δ 7.41-7.34 (m, aromatic hydrogens, 5H), 5.84-4.85 (o, aromatic hydrogens *p*-cymene - $NCH_2C\boldsymbol{H}$ - $NC\boldsymbol{H_2}CH$, 7H), 4.03 (s, $OC\boldsymbol{H_3}$, 3H), 3.24 (s, $NC\boldsymbol{H_3}$, 3H), 2.99 ($C\boldsymbol{H}(CH_3)_2$ *p*-cymene, 1H), 2.16 (s, $C\boldsymbol{H_3}$ *p*-cymene, 3H), 1.29 (m, $CH(C\boldsymbol{H_3})_2$ *p*-cymene, 6H).

^{13}C-NMR RANHC-V

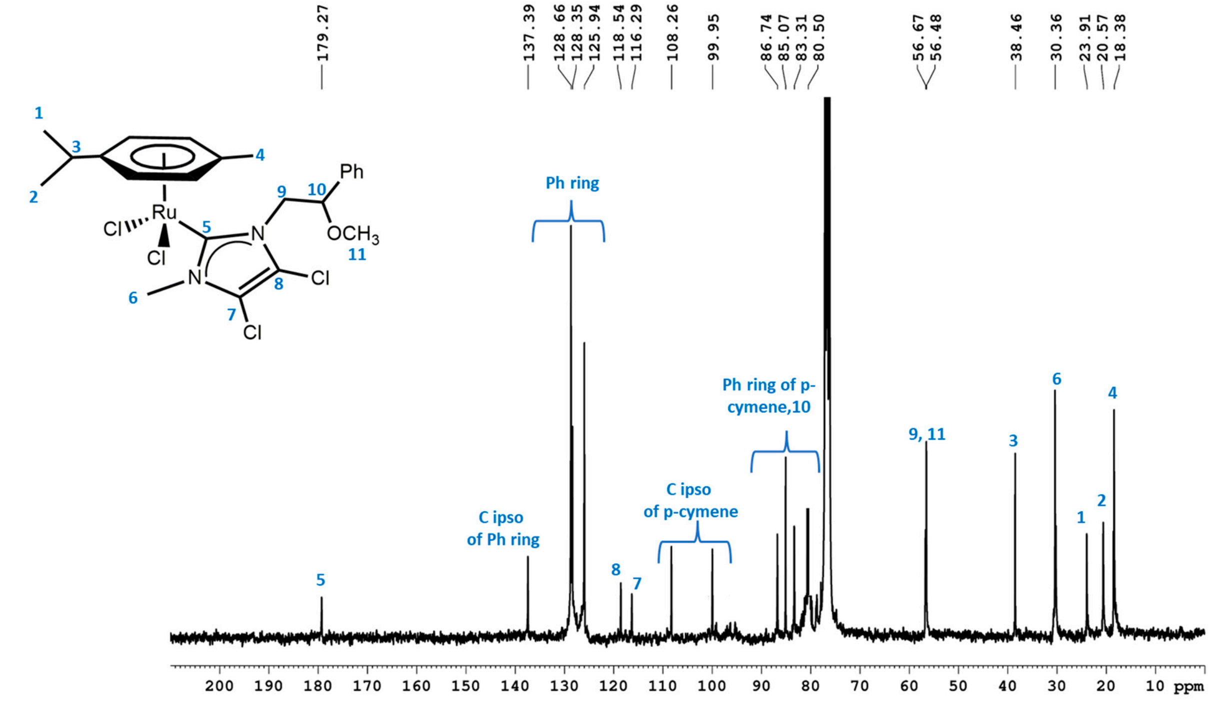

Figure S14: *^{13}C-NMR spectrum of* ***RANHC-V***

^{13}C-NMR (ppm, $CDCl_3$, 75 MHz): δ 179.27 (N***C***N), 137.39 (ipso carbon of aromatic ring), 128.66, 128.35, 125.94 (aromatic carbons),118.54, 116.29 (N***C***H***C***HN), 108.26, 99.95, 86.74 (aromatic carbons *p*-cymene), 85.07 (NCH_2***C***H), 83.31, 80.50 (aromatic carbons *p*-cymene), 56.67 (N***C***H2CH), 56.48 (O***C***H_3), 38.46(***C***H(CH_3)$_2$ *p*-cymene), 30.36 (N***C***H_3), 23.91, 20.57 (CH(***C***H_3)$_2$ *p*-cymene), 18.38 (***C***H_3 *p*-cymene).

ESI of RANHC-V

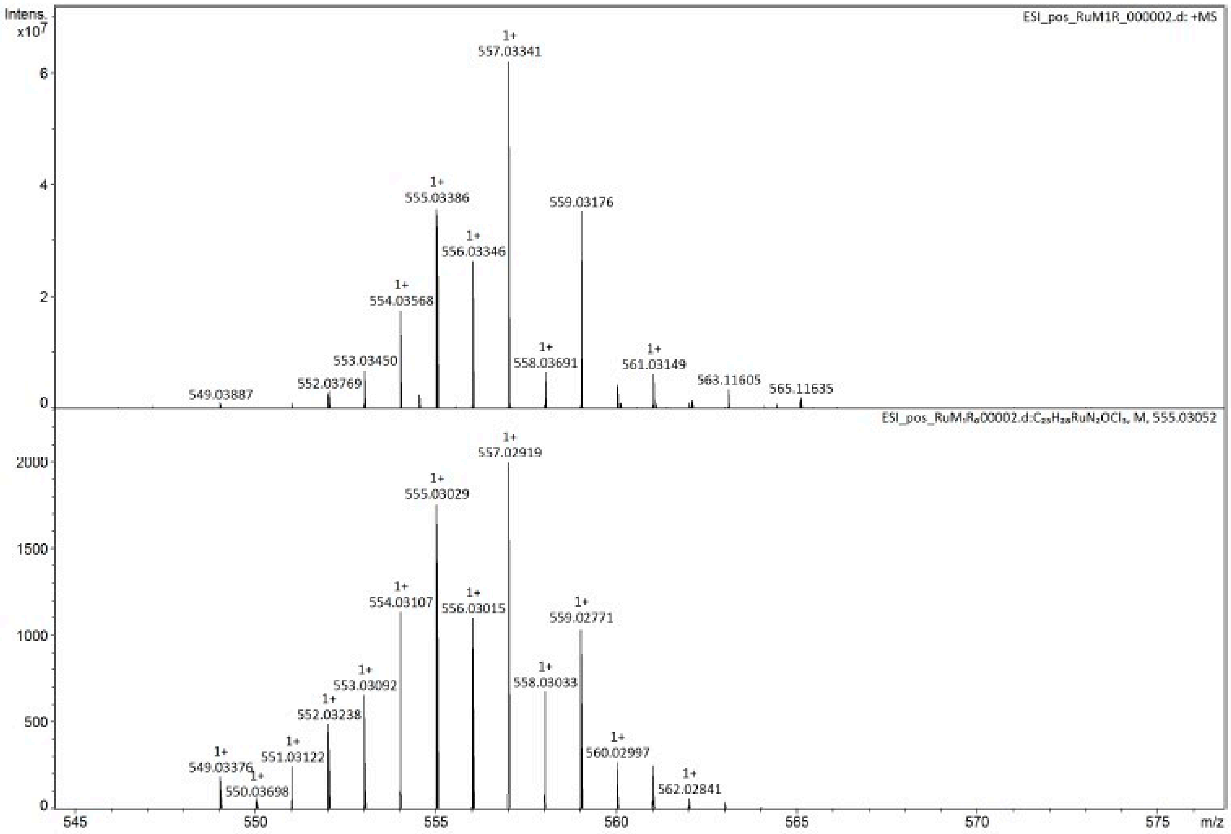

Figure S15: ESI of **RANHC-V**

^{1}H-NMR RANHC-VI

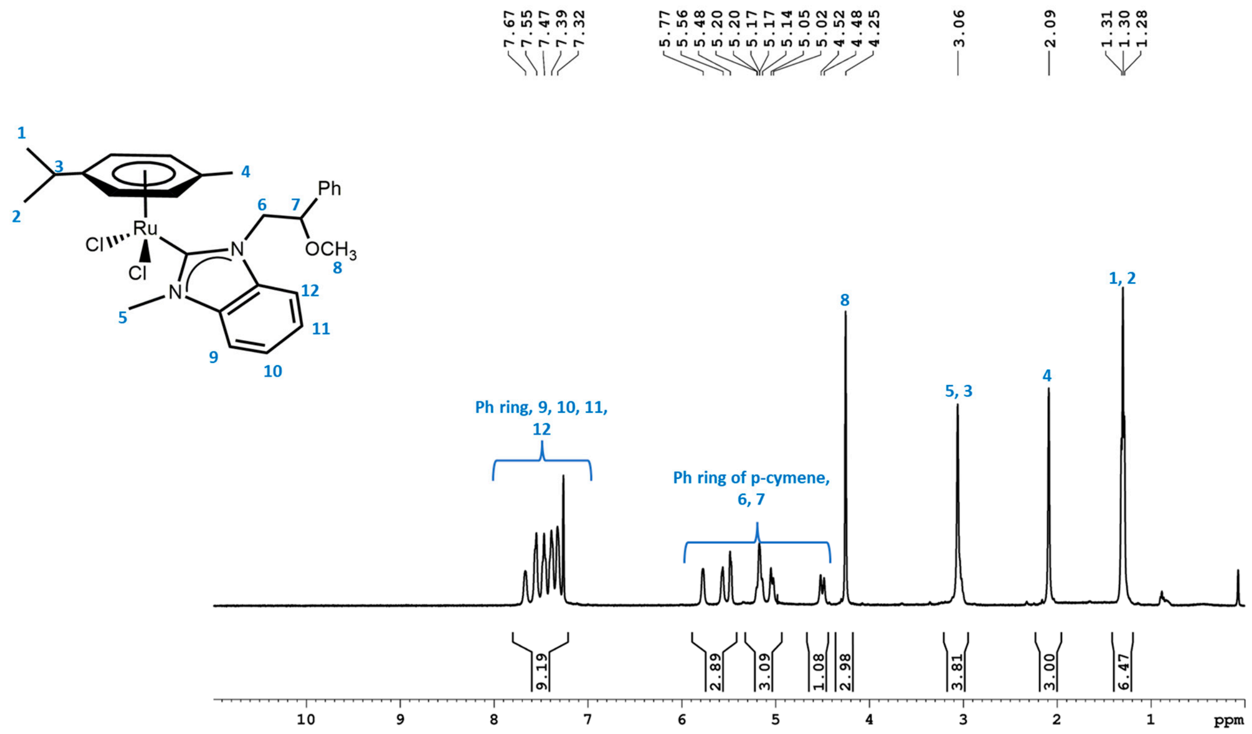

Figure S16: *^{1}H-NMR spectrum of* ***RANHC-VI***

^{1}H-NMR (ppm, $CDCl_3$, 400 MHz): δ 7.67-7.32 (m, hydrogens of aromatic rings, 9H), 5.77, 5.56 (br, aromatic hydrogens *p*-cymene, 2H), 5.48 (br, $NCH_2C\boldsymbol{H}$, 1H), 5.17 (m, $NC\boldsymbol{H}_2CH$, 2H), 5.05, 4.50 (d, hydrogens *p*-cymene, 2H), 4.25 (s, $OC\boldsymbol{H}_3$, 3H), 3.06 (o, $NC\boldsymbol{H}_3$ and $C\boldsymbol{H}(CH_3)_2$ *p*-cymene, 4H), 2.09 (s, $C\boldsymbol{H}_3$ *p*-cymene, 3H), 1.30 (m, $CH(C\boldsymbol{H}_3)_2$ *p*-cymene, 6H).

^{13}C-NMR RANHC-VI

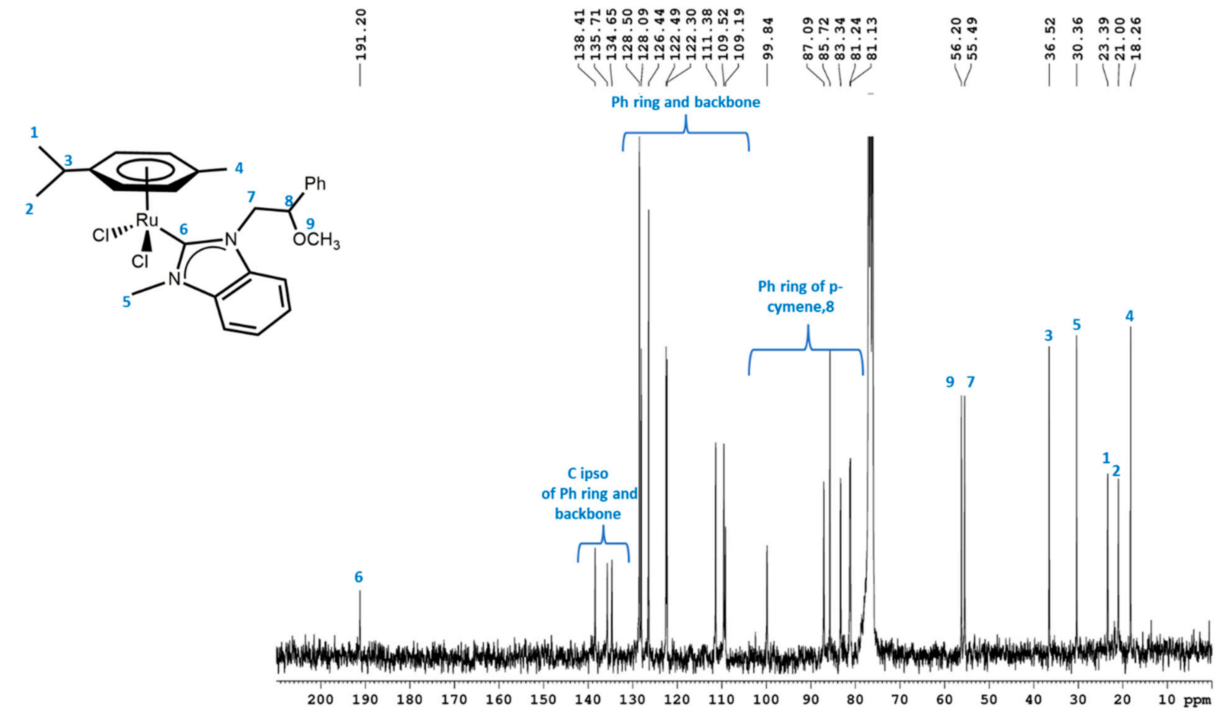

Figure S17: *^{13}C-NMR spectrum of* ***RANHC-VI***

^{13}C-NMR (ppm, $CDCl_3$, 75 MHz): δ 191.20 (N**C**N), 138.41 (ipso carbon of aromatic ring), 135.71, 134.65, 128.50, 128.09, 126.44, 122.49, 122.30, 111.38, 109.52 (aromatic carbons), 109.19, 99.84, 87.09, 85.72 (aromatic carbons *p*-cymene), 83.34 (NCH_2**C**H), 81.24,81.13 (aromatic carbons *p*-cymene), 56.20 (O**C**H_3), 55.49 (N**C**H_2CH), 36.52 (**C**H(CH_3)$_2$ *p*-cymene), 30.36 (N**C**H_3), 23.39, 21.00 (CH(**C**H_3)$_2$ *p*-cymene), 18.26 (**C**H_3, *p*-cymene).

ESI of RANHC-VI

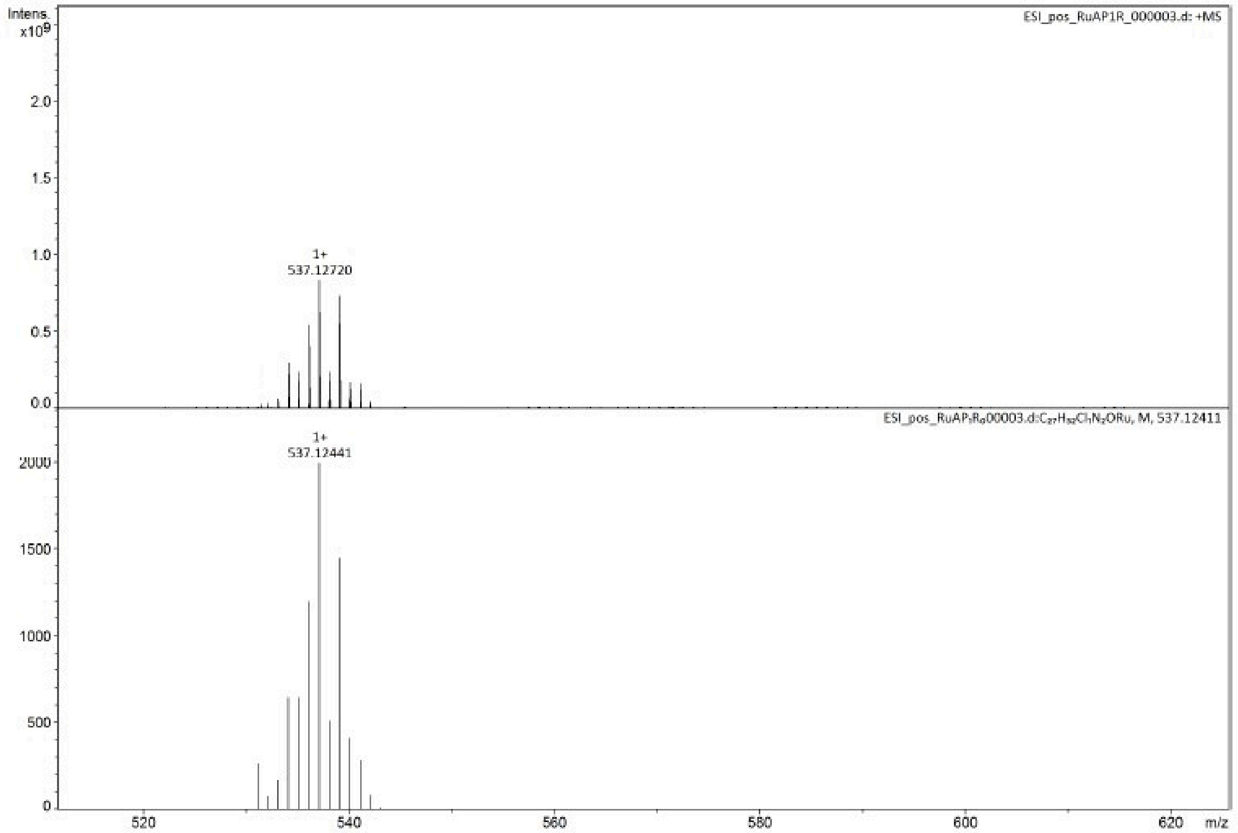

Figure S3**: ESI of **RANHC VI

^{1}H-NMR RANHC-II

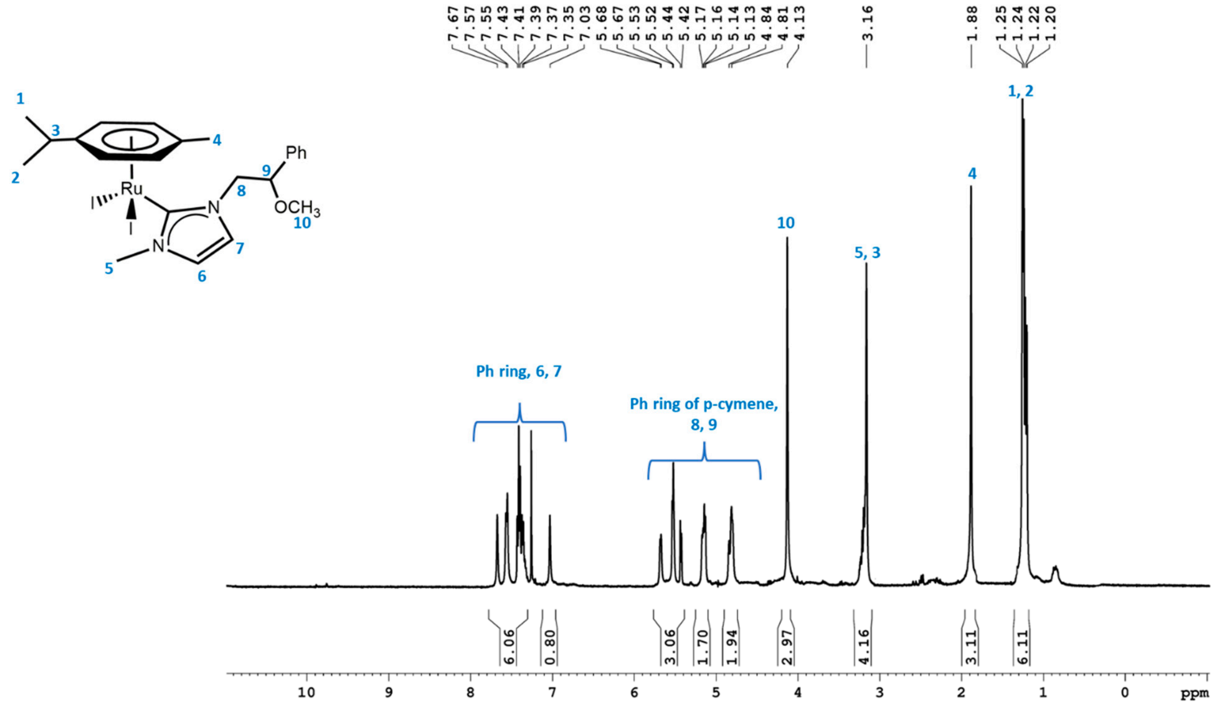

Figure S4**: ^{1}H-NMR spectrum of **RANHC-II

^{1}H-NMR (ppm, $CDCl_3$, 400 MHz): δ 7.67-7.03 (m, aromatic hydrogens and NC***H***C***H***N, 7H), 5.68-5.42 (br, aromatic hydrogens of *p*-cymene, 3H), 5.17 (o, aromatic hydrogens of *p*-cymene and NCH_2C***H***OCH_3, 2H), 4.81 (m, NC***H***$_2$$CHOCH_3$, 2H), 4.13 (s, OC***H***$_3$, 3H), 3.16 (o, NC***H***$_3$ and C***H***$(CH_3)_2$ *p*-cymene, 4H), 1.88 (s, C***H***$_3$ *p*-cymene, 3H), 1.22 (m, CH(C***H***$_3$)$_2$ *p*-cymene, 6H).

^{13}C-NMR RANHC-II

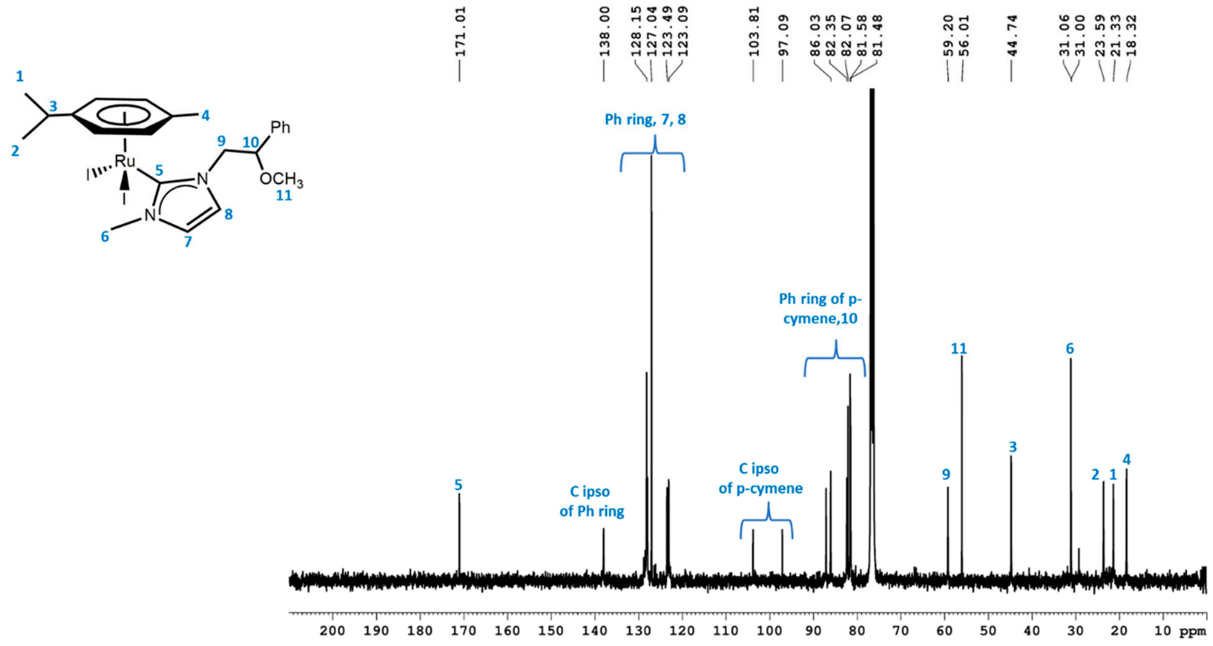

Figure S20: *^{13}C-NMR spectrum of* ***RANHC-II***

^{13}C-NMR (ppm, $CDCl_3$, 100 MHz): δ 171.01 (N***C***N), 138.00 (ipso carbon of aromatic ring), 128.15, 127.04 (aromatic carbons), 123.49,123.09 (N***C***H***C***HN), 103.81, 97.09 (aromatic carbons *p*-cumene), 86.03 (NCH$_2$***C***H), 82.35, 82.07, 81.58, 81.48 (aromatic carbons *p*-cymene), 59.20 (N***C***H$_2$CH), 56.01 (O***C***H$_3$), 44.74 (***C***H(CH$_3$)$_2$ *p*-cymene), 31.06 (N***C***H$_3$), 23.59, 21.33 (CH(***C***H$_3$)$_2$ *p*-cymene), 18.32 (***C***H$_3$ *p*-cymene).

ESI of RANHC-II

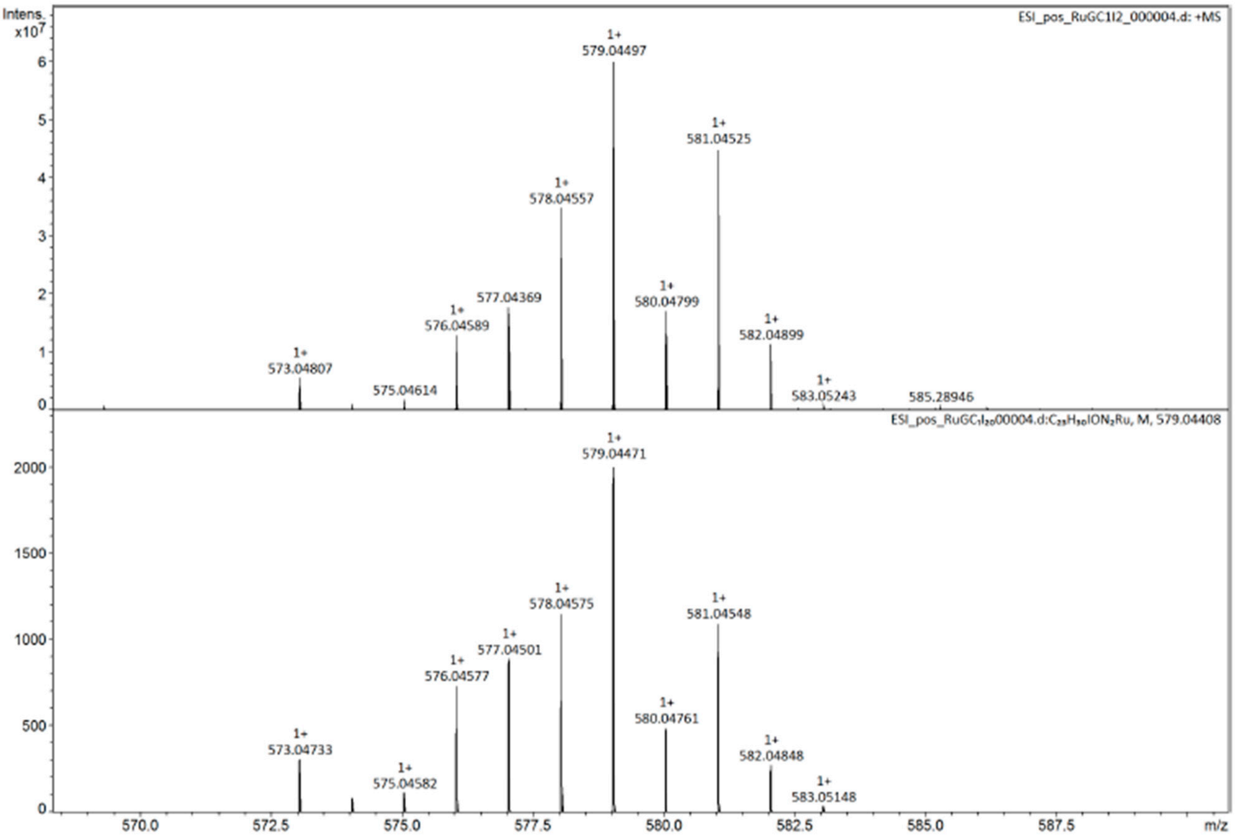

Figure S21: *ESI of* ***RANHC-II***

^{1}H-NMR RANHC-III

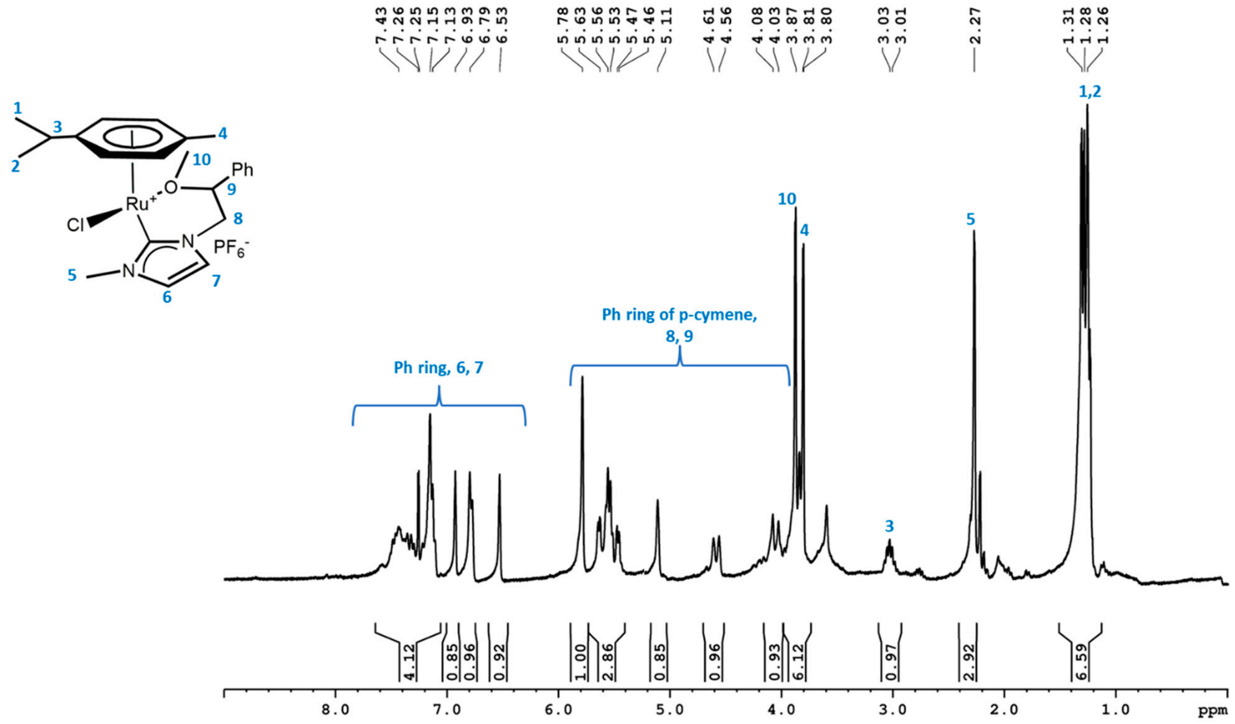

Figure S22**: ^{1}H-NMR spectrum of **RANHC-III

^{1}H-NMR (ppm, $CDCl_3$, 300 MHz): δ 7.43-6.53(m, aromatic hydrogens and NC***H***C***H***N, 7H), 5.78-4.08 (o, aromatic hydrogens of *p*-cymene - NC***H***$_2$CHOCH$_3$ - NCH$_2$C***H***OCH$_3$, 7H), 3.87 (s, OC***H***$_3$, 3H), 3.80 (s, C***H***$_3$ *p*-cymene, 3H), 3.02 (m, C***H***(CH$_3$)$_2$ *p*-cymene, 4H), 2.27 (s, NC***H***$_3$, 3H), 1.28 (m, CH(C***H***$_3$)$_2$ *p*-cymene, 6H).

^{13}C-NMR RANHC-III

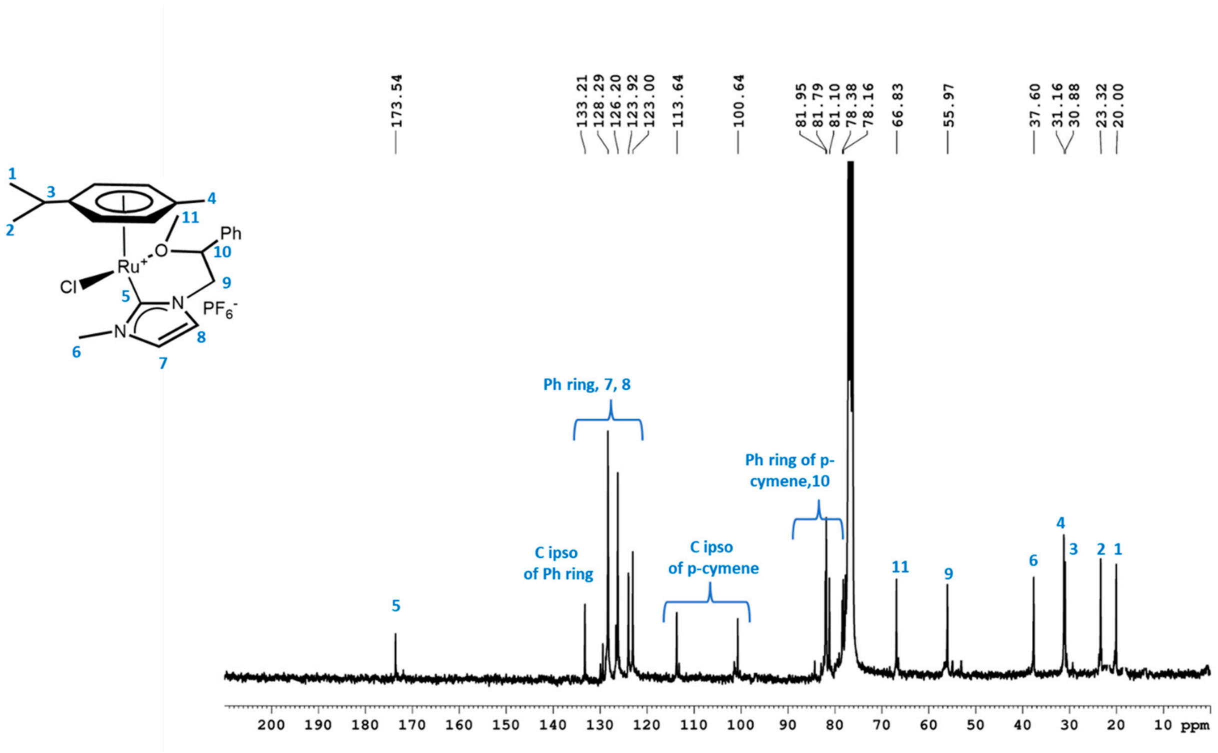

Figure S23: *^{13}C-NMR spectrum of* ***RANHC-III***

^{13}C-NMR (ppm, $CDCl_3$, 62.5 MHz): δ 173.54 (N***C***N), 133.21 (ipso carbon of aromatic ring), 128.29, 126.20 (aromatic carbons), 123.92,123.00 (N***C***H***C***HN), 113.64, 100.64 (aromatic carbons *p*-cumene), 81.95 (aromatic carbon *p*-cymene), 81.79 (NCH_2***C***H), 81.10, 78.38, 78.16 (aromatic carbon *p*-cymene), 66.83 (O***C***H_3), 55.97 (N***C***H_2CH), 37.60 (***C***H(CH_3)$_2$ *p*-cymene), 31.16 (N***C***H_3), 30.88 (***C***H_3 *p*-cymene). 23.32, 20.00 (CH(***C***H_3)$_2$ *p*-cymene).

^{31}P-NMR RANHC-III

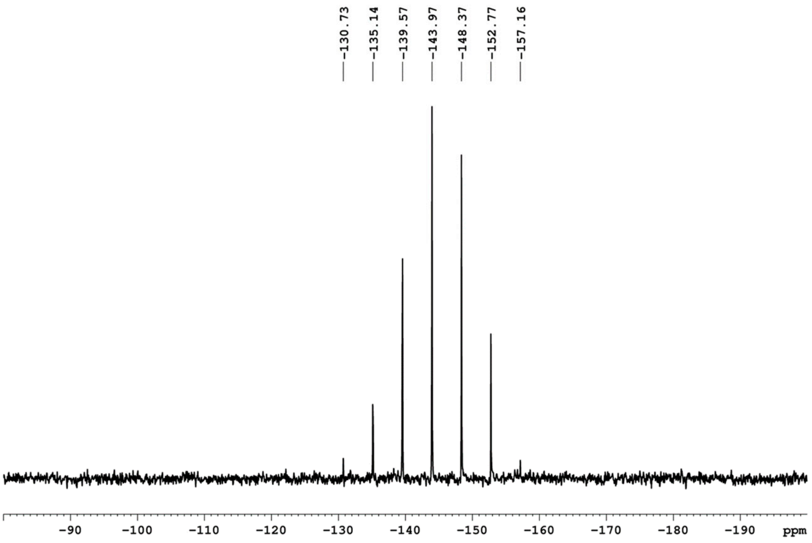

Figure S24: *^{31}P-NMR spectrum of* ***RANHC-III***

^{31}P-NMR (ppm, $CDCl_3$, 161.97 MHz): δ -143.97 (m)

^{19}F-NMR RANHC-III

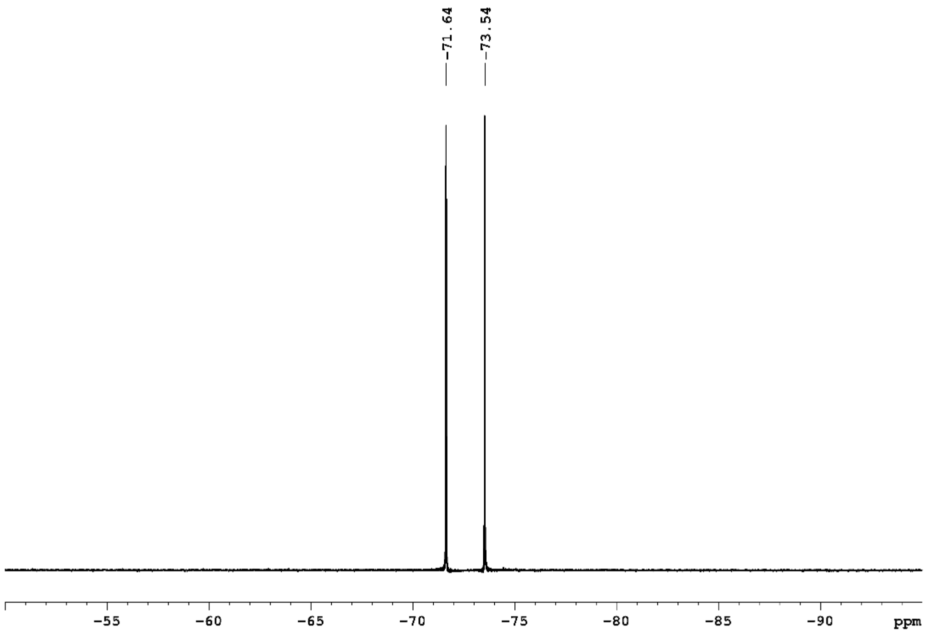

Figure S25:** ^{19}F-NMR spectrum of* ***RANHC-III

^{19}F-NMR (ppm, $CDCl_3$, 376 MHz): δ -71.64, -73.54.

ESI of RANHC-III

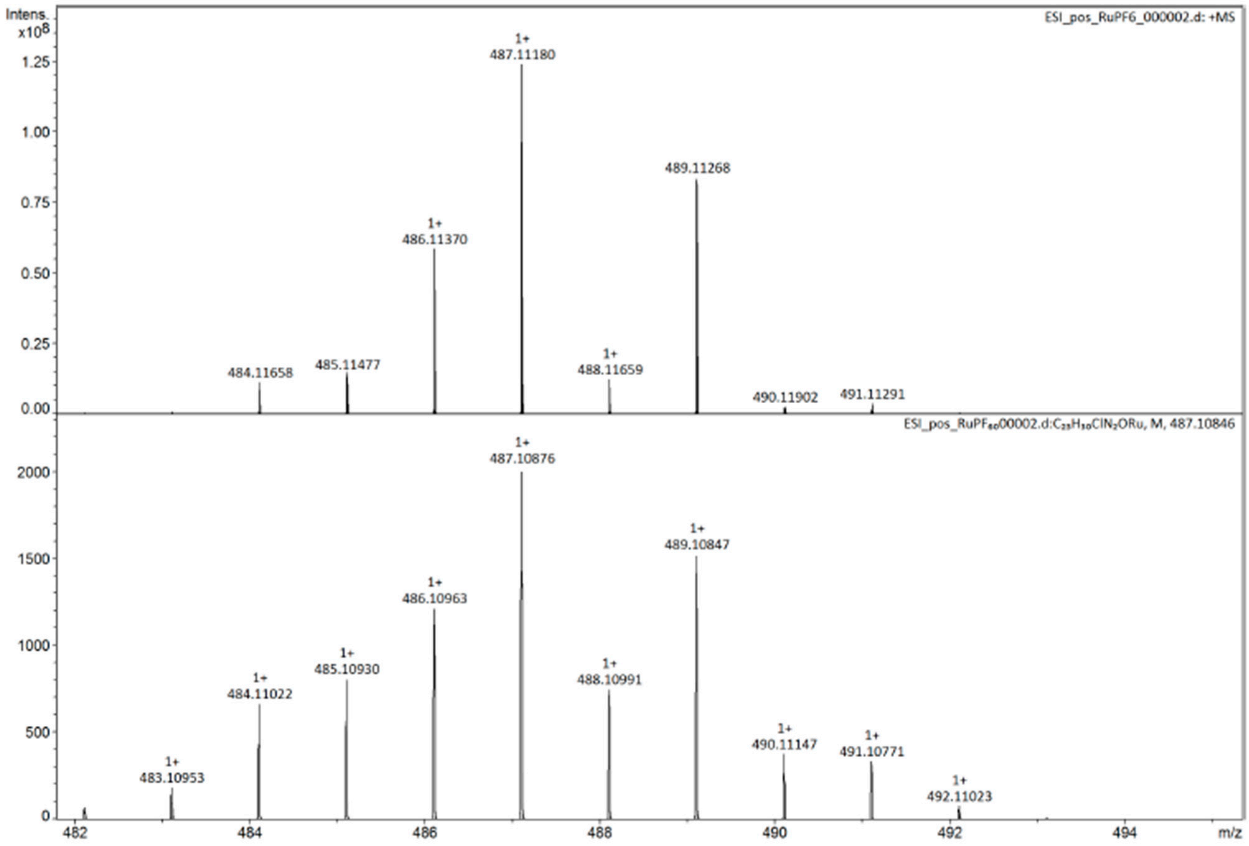

__Figure S26__: ESI of __RANHC-III__

^{1}H-NMR RANHC-IV

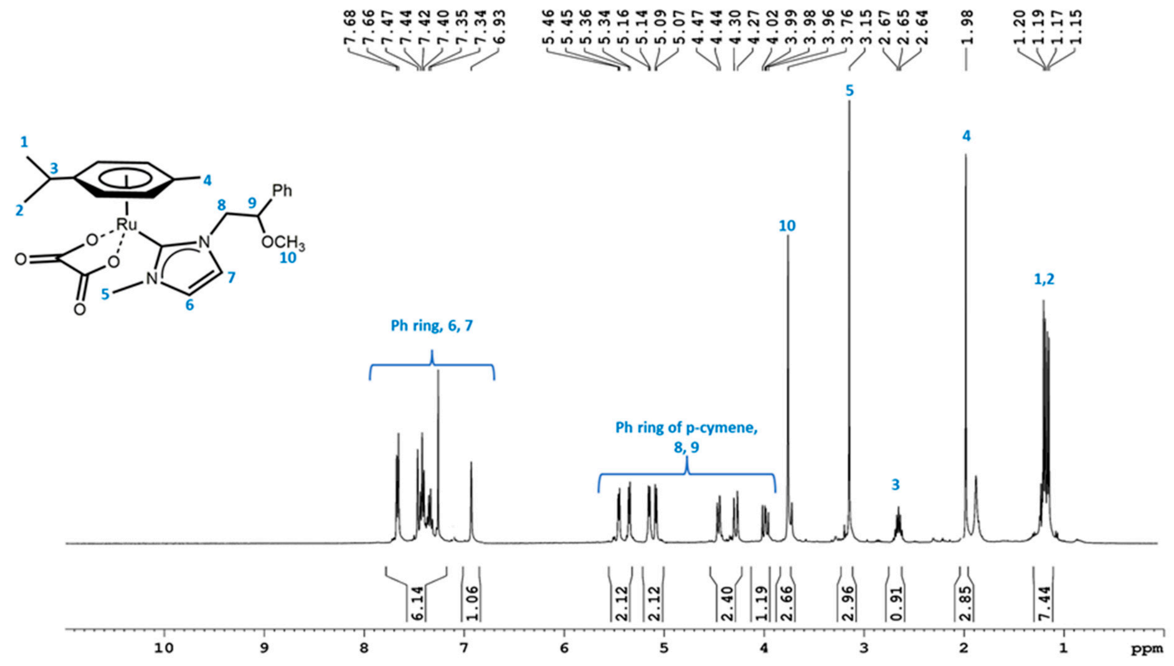

Figure S27: *^{1}H-NMR spectrum of* ***RANHC-IV***

^{1}H-NMR (ppm, $CDCl_3$, 400 MHz): δ 7.68-6.93 (m, aromatic hydrogens and NC***H***C***H***N, 7H), 5.46-5.34 (dd, aromatic hydrogens of *p*-cymene, 2H), 5.16-5.07 (dd, aromatic hydrogens of *p*-cymene, 2H), 4.47-4.27 (dd, NC***H*2**CH, 2H), 3.98 (t, NCH_2C***H***, 1H), 3.76 (s, OC***H***$_3$, 3H), 3.15 (s, NC***H***$_3$, 3H), 2.65 (m, C***H***(CH3)$_2$ *p*-cymene, 1H), 1.98 (s, C***H***$_3$ *p*-cymene, 3H), 1.18 (m, CH(C***H***$_3$)$_2$ *p*-cymene, 6H).

^{13}C-NMR RANHC-IV

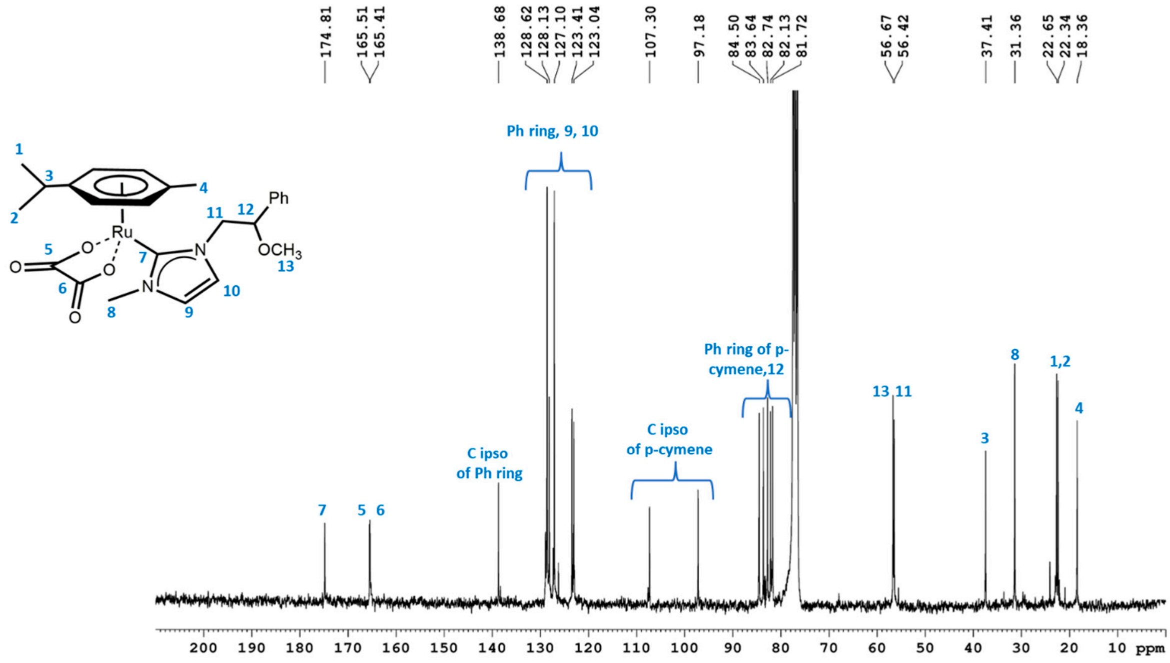

Figure S28: *^{13}C-NMR spectrum* ***of RANHC-IV***

^{13}C-NMR (ppm, $CDCl_3$, 62.5 MHz): δ 174.81 (N***C***N), 165.51, 165.41 (***C***=O, oxalyl group), 138.68 (ipso carbon of aromatic ring), 128.68, 128.13,127.10 (aromatic carbons), 123.41,123.04 (N***C***H***C***HN), 107.30, 97.18 (aromatic carbons *p*-cymene), 84.50(NCH_2***C***H), 83.64, 82.74, 82.13, 81.72 (aromatic carbons *p*-cymene), 56.67 (O***C***H_3), 56.42 (N***C***H_2CH), 37.41 (***C***H(CH_3)$_2$ *p*-cymene), 31.36 (N***C***H_3), 22.65, 22.34 (CH(***C***H_3)$_2$ *p*-cymene), 18.36 (***C***H_3 *p*-cymene).

ESI of RANHC-IV

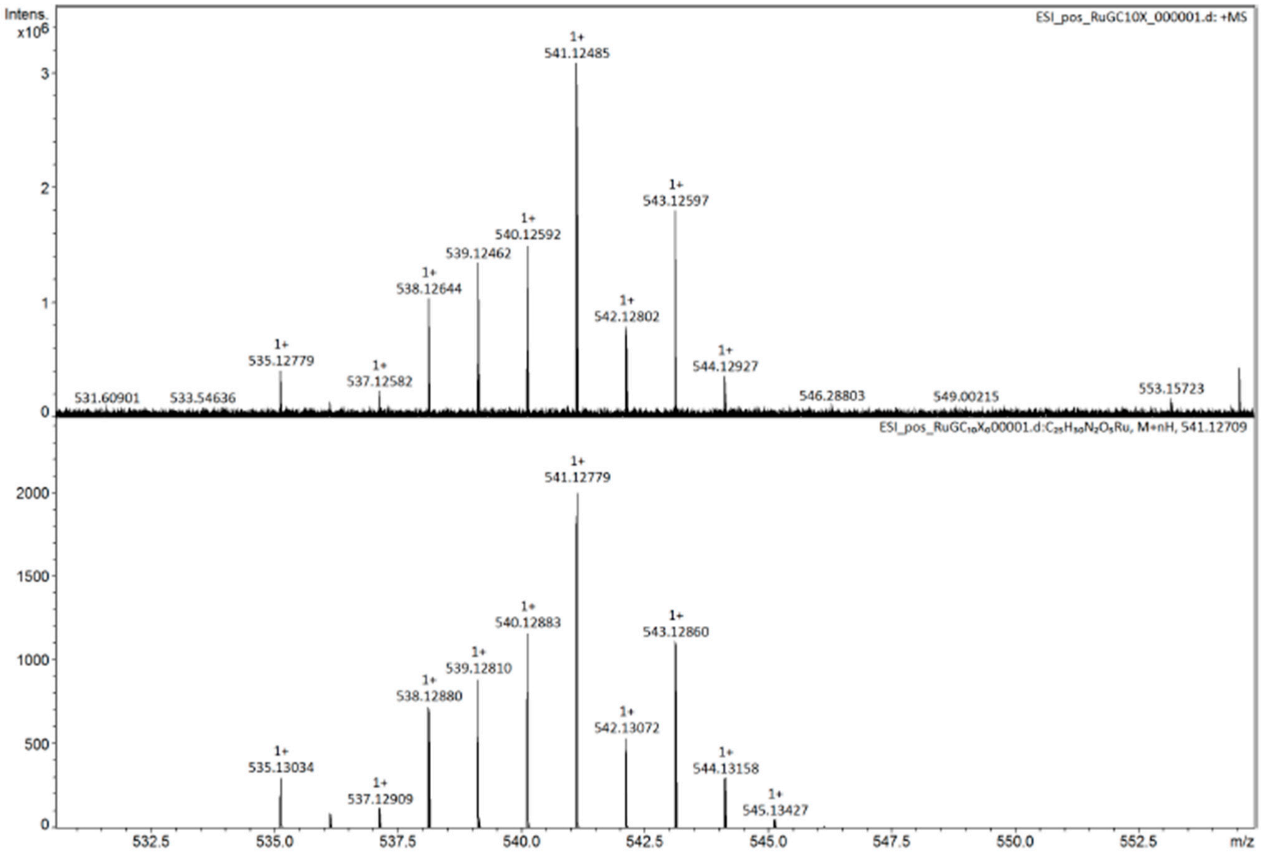

Figure S29: *ESI of* ***RANHC-IV***

Table S1: Anticancer activity of the studied Ru-NHC complexes (**RANHC-I-VI**), expressed as IC_{50} values ± S.D. μM and μg/mL, against different cell lines.

Compounds	MDA-MB-231		MCF-7		SH-SY5Y		MCF-10A		BALB/3T3	
	**μM**	**μg/mL**	**μM**	**μg/mL**	**μM**	**μg/mL**	**μM**	**μg/mL**	**μM**	**μg/mL**
**RANHC-I**	>100	>52.25	>100	>52.25	90.05±0.9	47.05±0.9	>100	>52.25	>100	>52.25
**RANHC-II**	>100	>70.54	>100	>70.54	>100	>70.54	>100	>70.54	>100	>70.54
**RANHC-III**	>100	>63.20	>100	>63.20	>100	>63.20	>100	>63.20	>100	>63.20
**RANHC-IV**	>100	>53.96	>100	>53.96	88.89±0.9	47.96±0.9	>100	>53.96	>100	>53.96
**RANHC-V**	24.14±0.7	14.28±0.7	26.05±0.9	15.41±0.9	48.43±0.8	28.64±0.8	79.47±1.2	46.99±1.2	>100	>59.14
**RANHC-VI**	40.57±1.1	23.23±1.1	54.75±1.1	31.35±1.1	66.86±0.8	38.28±0.8	90.72±1.2	51.94±1.2	39.09±1.1	22.38±1.1
**Cisplatin**	32.15±1.0	9.68±1.0	26.19±1.1	7.89±1.1	18.75±0.9	5.65±0.9	80.24±0.8	24.16±0.8	21.57±1.2	6.49±1.2

(Table header spanning all cell lines: **IC_{50}**)

Table S2. MIC results of the Ru-NHC complexes (**RANHC-I-VI**), expressed in μM and μg/mL.

Ru-NHC complexes	*E. coli*[b]		*S. aureus*[b]		*E. faecalis*[b]	
	**μM**	**μg/mL**	**μM**	**μg/mL**	**μM**	**μg/mL**
**RANHC-I**	95.70	50	47.85	25	95.70	50
**RANHC-II**	70.88	50	35.44	25	70.88	50
**RANHC-III**	39.56	25	39.56	25	79.12	50
**RANHC-IV**	92.66	50	46.33	25	92.66	50
**RANHC-V**	84.55	50	42.27	25	118.37	70
**RANHC-VI**	43.67	25	43.67	25	87.33	50

(Table header spanning all organisms: **M.I.C. [a]**)

Table S3. Radical scavenging ability against DPPH and ABTS radicals, expressed as IC_{50} ± SD μM and μg/mL, of Ru-NHC complexes and standard drug (Trolox).

Compounds	IC_{50} ABTS		IC_{50} DPPH	
	µM	µg/mL	µM	µg/mL
**RANHC-I**	13.52±0.7	7.06±0.7	369.6±1.1	193.11±1.1
**RANHC-II**	16.05±0.7	11.32±0.7	214.8±1.1	151.52±1.1
**RANHC-III**	5.53±1.1	3.49±1.1	44.19±1.2	27.93±1.2
**RANHC-IV**	8.57±1.1	4.62±1.1	512.3±0.8	276.43±0.8
**RANHC-V**	11.36±0.8	6.72±0.8	246.2±1.2	145.60±1.2
**RANHC-VI**	17.21±1.1	9.85±1.1	161.6±1.0	92.52±1.0
**Trolox**	92.30±0.9	23.10±0.9	99.91±0.9	25.01±0.9
